# Chemical Composition, Biological and Cytotoxic Activities of Plant Extracts and Compounds Isolated from *Ferula lutea*

**DOI:** 10.3390/molecules19032733

**Published:** 2014-02-26

**Authors:** Mansour Znati, Hichem Ben Jannet, Sylvie Cazaux, Jalloul Bouajila

**Affiliations:** 1 Laboratoire des IMRCP UMR CNRS 5623, Faculté de pharmacie de Toulouse, Université de Toulouse, Université Paul-Sabatier, 118 route de Narbonne, Toulouse F-31062, France; 2 Laboratoire de Chimie Hétérocyclique, Produits Naturels et Réactivité (CHPNR), Equipe Chimie Médicinale et Produits Naturels, Département de Chimie, Faculté des Sciences de Monastir, Université de Monastir, Avenue de l’Environnement, Monastir 5019, Tunisia

**Keywords:** *Ferula lutea*, (+)-*Z*-deltoin, (−)-*E*-deltoin, antioxidant, antiacetylcholinesterase, antimicrobial, antidiabetic, cytotoxic, allelopathic

## Abstract

The present work describes the phytochemical study on *Ferula lutea* flowers. Total phenolics and flavonoids of the *n*-butanol and ethyl acetate extracts were quantified (phenolics [40.68–52.29 mg gallic acid equivalent/g of dry weight], flavonoids [12.38–14.72 mg quercitin/g dry weight]). Two diastereoisomers were isolated and identified using spectroscopic techniques (1D, 2D NMR and GC-MS). The extracts and diastereoisomers were tested for antioxidant, antiacetylcholinesterase, antimicrobial, antidiabectic, cytotoxic (leukemia cell line) activities and allelopathic potentialities. The strongest antioxidant activity was obtained for the ethyl acetate extract (IC_50_ = 12.8 ± 1.29 µg/mL). The two extracts exhibited high antidiabetic activity (54.1 and 52.1% at 40 µg/mL).

## 1. Introduction

Plants still serve as a far reaching source of innovative and original compositions on the structural level. These new compounds serve as a clue for the discovery of new medicines, herbal and practical foods. There is an escalating interest in naturally occurring antioxidants to replace synthetic counterparts used for food conservation, flavoring, and cosmetics, as well as in health care. 

The genus *Ferula* (family *Apiaceae*) comprises 170 species [1], mostly growing in arid regions of temperate Eurasia, in the Canary Islands and in North Africa. The center of diversity of the genus is situated in W. and C. Asia [2]. In Tunisia, only four species of the *Ferula* genus have been identified: *F. communis*, *F. lutea, F. tunetana* and *F. tingitana* [3]. Several species of this genus have been used in folk medicine in several countries. The gum resins of the roots from several *Ferula* species are reported to be used for stomach disorders, rheumatism, headache, arthritis, and dizziness [4,5]. Some species are used in traditional foods as well as in traditional medicine as treatments for skin infections [6] and diabetes, and prevent convulsion and hysteria [7]. It is known that the genus *Ferula* contains a variety of coumarins [8] and sesquiterpenes [9]. Chemical investigations on *F. communis* also led to the isolation of coumarins [10,11] and sesquiterpenes [11]. Various biological activities have been reported for the essential oils of some species of *Ferula*, such as antioxidant [12], cytotoxicity [13] and antibacterial properties [14]. Natural products bearing coumarin moieties have shown a variety of biological activities [15,16]. Coumarins such as (‒)-deltoin are hepatoprotective and show TNF-α inhibitory activity [16]. The roots of *F. lutea* were the subject of a previous phytochemical investigation, undertaken in our laboratory, leading to the isolation of new dihydrofuranocoumarins as two inseparable isomers, (‒)-5-hydroxyprantschimgin and (‒)-5-hydroxydeltoin, together with eight known compounds, (‒)-prantschimgin, (‒)-deltoin, psoralen, xanthotoxin, umbelliferone, caffeic acid, β-sitosterol and stigmasterol [17]. The essential oil obtained from *F. lutea* flowers has recently been shown to contain various terpenoidal compounds [18].

In this work, we report the, antioxidant, antiacetylcholinesterase, antimicrobial, antidiabetic, allelopathic and cytotoxic activities of the ethyl acetate and the *n*-butanol extract of *F. lutea* flowers and the isolation, and identification of two diastereoisomers in the mixture. This is the first time that extracts and compounds **1a** and **1b** were evaluated for biological activities.

## 2. Results and Discussion

### 2.1. Extraction Yields

Extraction yields of *F. lutea* flowers are presented in Table 1. The *n*-butanol extract had the highest yield (3.79%), while the ethyl acetate one gave 0.96%. No data on extraction yields of this plant have been found in the literature.

**Table 1:** Yields and chemical composition of *F. lutea* flowers extracts.

Extracts	Yield (%)	Phenolics (GAE) ^a^	Flavonoids (QE) ^a^
Ethyl acetate	0.96	40.7 ± 0.2	12.4 ± 0.1
*n*-Butanol	3.79	52.3 ± 0.4	14.7 ± 0.1

GAE: gallic acid equivalents. QE: quercetin equivalent. ^a^: mg/g dry weight.

### 2.2. Isolation and Structure Elucidation of Compounds **1a** + **1b**

**Figure 1:**
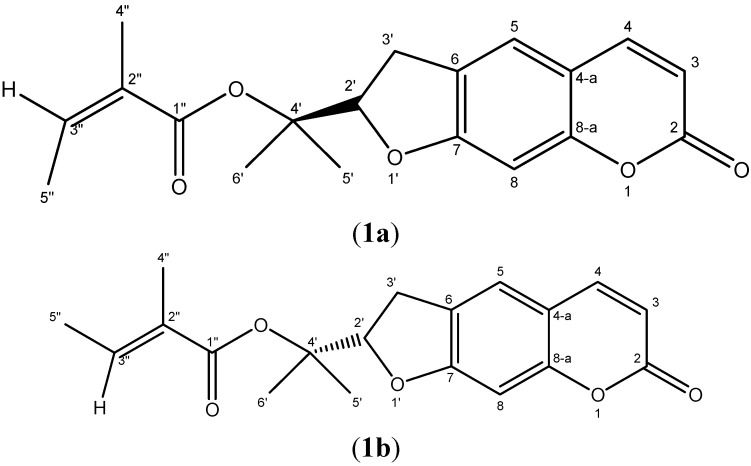
Structure of (+)-*Z*-deltoin (**1a**, major compound) and (−)-*E*-deltoin (**1b**, minor compound).

The mixture of compounds **1a** and **1b** was isolated as a yellow oil and it showed on TLC a spot featuring a characteristic blue fluorescence under UV light. EI GC-MS analysis of the mixture gave for both a molecular ion peak [M]^+^ at *m/z* 328 indicating a common molecular formula of C_19_H_20_O_4_. The structure of **1a** and **1b** (Figure 1) were elucidated on the basis of the ^1^H- and ^13^C-NMR spectral data (Table 2).

**Table 2:** ^1^H- and ^13^C-NMR spectroscopic data of the two diastereoisomers (**1a** and **1b**).

Position	1a			1b		
	δ_H_	Multiplicity *J* (Hz)	δ_C_	δ_H_	Multiplicity *J* (Hz)	δ_C_
2	-	-	160.99	-	-	161.51
3	6.17	d (9.6)	111.64	6.20	d (9.6)	112.26
4	7.57	d (9.6)	143.28	7.56	d (9.6)	143.78
5	7.19	s	122.76	7.14	s	123.20
6	-	-	124.04	-	-	124.50
7	-	-	162,89	-	-	163.44
8	6.69	s	97.26	6.76	s	97.84
4-a	-	-	112.11	-	-	112.21
8-a	-	-	155.18	-	-	155.75
2'	5.02	t (9)	88.67	5.12	m	88.91
3'-a	3.10–3.30	m		3.10–3.30	m	
3'-b	3.10–3.30	m	29.06	3.10–3.30	m	29.61
4'	-	-	81.49	-	-	82.04
5'	1.58	s	20.94	1.37	s	20.58
	1.59	s	21.73	1.39	s	21.47
1''	-	-	166.57	-	-	168.67
2''	-	-	128.11	-	-	127.33
3''	5.95	qq (7.2; 1.5)	137.28	6.09	qq (7.2; 1.2)	137.74
4''	1.63	m	19.56	1.63	m	20.09
5''	1.87	m	15.13	1.85	m	15.64

**Figure 2:**
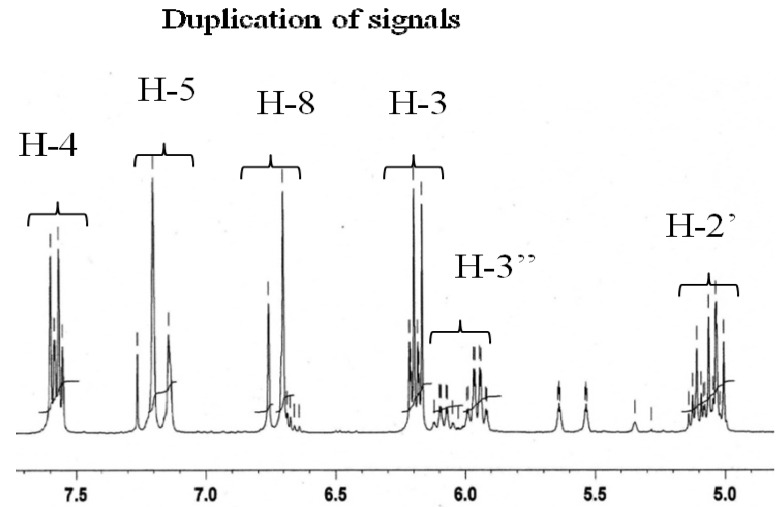
Duplication of signals in ^1^H-NMR of the mixture of compounds **1a** and **1b**.

The ^1^H-NMR spectrum showed the duplication of all the proton signals of deltoin (Figure 2) [19]. This spectrum showed two signals at δ_H_ 6.17 (1H, d, *J* = 9.6 Hz) and δ_H_ 7.57 (1H, d, *J* = 9.6 Hz) as AB-type signals, attributable to H-3 and H-4 in **1a**, respectively. A second AB-type spin system was also observed at δ_H_ 6.20 (1H, d, *J* = 9.6 Hz) and δ_H_ 7.56 (1H, d, *J* = 9.6 Hz), corresponding to H-3 and H-4 in **1b**, respectively. 

The same spectrum showed two pair of singlets at δ_H_ 6.69 (1H, s, **1a**) and δ_H_ 7.19 (1H, s, **1a**) and at δ_H_ 6.76 (1H, s, **1b**) and δ_H_ 7.14 (1H, s, **1b**) corresponding to the aromatic protons H-8 and H-5, respectively. The observation in the same spectrum of a triplet at δ_H _5.02 (1H, t, *J* = 9 Hz, H-2', **1a**) and a multiplet at δ_H _5.12 (1H, m, H-2', **1a**) a multiplet at δ_H_ 3.10–3.30 (4H, m, H-3'a,b, **1a** and **1b**) suggested the presence of the dihydrofuranic moiety in **1a** and **1b** [17,19,20,21,22,23,24]. These data and the observed duplication of all the signals are in good concordance with the dihydrofuranocoumarin structure of **1a** and **1b**. The two methyl groups attached at C-4' in both **1a** and **1b** were identified according to the two pair of singlets at δ_H_ 1.58 (3H, s, H-5') and 1.59 (3H, s, H-6') and at δ_H_ 1.37 (3H, s, H-5') and 1.39 (3H, s, H-6'), respectively. The other signals at δ_H_ 5.95 (1H, qq, *J* = 7.2; 1.5 Hz, H-3'', **1a**), at δ_H_ 1.63 (3H, m, H-4'', **1a**), at δ_H_ 1.87 (3H, m, H-5'', **1a**), at δ_H_ 6.09 (1H, qq, *J* = 7.2; 1.2 Hz, H-3'', **1b**), at δ_H_ 1.63 (3H, m, H- 4'', **1b**), at δ_H_ 1.85 (3H, m, H-5'', **1b**) coincide with the spectral data of an angeloyl moiety in **1a** and **1b** with two different configurations (Figure 1).

The^ 13^C-NMR spectrum showed the duplication of most signals including those attributed to the common dihydrofuranocoumarin system and those of the angeloyl moiety confirming that the analyzed sample is a mixture of two diastereoisomeric forms **1a** and **1b** (Table 2).

The stereochemical proposal for carbon C-2' in **1a** has been attributed based on comparison of the chemical shift of H-2' (δ_H_ 5.09) to the literature data [20]. On the other hand, the (*Z*) configuration of the double bond in the angeloyl fragment in **1a** was evidenced from the NOESY spectrum showing the dipolar interaction of the ethylenic proton H-3'' (δ_H_ 5.95, qq, *J_1_* = 7.2 Hz, *J_2_* = 1.5 Hz) with both methyl groups (CH_3_)-4'' (δ_H_ 1.63, m) and (CH_3_)-5'' (δ_H_ 1.87, m), whereas, the (*E*) configuration of the same double bond in the angeloyl moiety in **1b** was proved by the unique *nOe* between H-3" (δ_H_ 6.09, qq, *J* = 7.2; 1.2 Hz), and H-5" (δ_H_ 1.85, m) revealed from the same NOESY spectrum.

The various plants and organs containing compounds **1a** and **1b** are grouped in Table 3. Compound **1a** was never previously isolated from any *Ferula* species, but it was previously isolated from *Seseli peucedanoides* (roots) and *Heracleum candolleanum* (seeds or roots), while compound **1b** was isolated from *Peucedanum japonicum* (roots), *Seseli resinosum* (roots) and *Ferula lutea* (roots). Compounds **1a** and **1b** were identified for the first time in the *F. lutea* flowers. 

**Table 3:** Various plants and organs containing compounds **1a** and **1b**.

Compound	Plant	Reference
**1a**	*Seseli peucedanoides* (roots)	[21]
	*Heracleum candolleanum* (seeds or roots)	[22]
**1b**	*Ferula lutea* (root)	[17]
	*Peucedanum japonicum* (root)	[23]
	*Seseli resinosum* (root)	[24]

### 2.3. Phenolics and Flavonoids Content

The amount of total phenolics and flavonoids content in the extracts of *F. lutea* flowers are shown in Table 1. The *n*-butanol extract had the highest quantity (52.3 ± 0.4 mg GAE/g of dry weight) followed by the ethyl acetate extract (40.7 ± 0.2 mg GAE/g of dry weight). The flavonoids content of the *n*-butanol extract was the highest (14.7 mg QE/g dry weight) while the ethyl acetate extract had 12.4 ± 0.1 mg QE/g dry weight. The phenolics and flavonoids content of *F. lutea* has not been reported in the literature. The study of other species and organs (*F. assafoetida*, aerial parts) showed that the total phenolics were 94.8 ± 5.9 mg GAE/g of dry weight and total flavonoids were 90.9 ± 6.9 mg QE/g dry weight [25].

### 2.4. Antioxidant Activity

The antioxidant activity of the extracts assayed using two methods (DPPH and ABTS) is presented in Table 4. By the DPPH test, the best result was obtained with the ethyl acetate extract (IC_50_ = 12.8 ± 1.3 µg/mL) followed by the *n*-butanol extract (IC_50_ = 26.0 ± 1.8 µg/mL). The two extracts have good antioxidant activity compared to the positive control, L-ascorbic acid (Vit C, IC_50_ = 4.4 ± 0.1 µg/mL). The ABTS assay confirmed the DPPH assay results, with the ethyl acetate showing an IC_50_ = 184.0 ± 7.0 µg/mL and therefore better antioxidant activity than the *n*-butanol extract (IC_50_ = 300.0 ± 5.0 µg/mL). The antioxidant activity (DPPH and ABTS) of *F. lutea* extracts has not been cited in the literature before. For this genus, only *F. gummosa* Boiss roots [26] was evaluated for antioxidant activity with the DPPH assay giving an IC_50_ = 579.6 ± 19.4 µg/mL.

**Table 4:** Antioxidant, antiacetylcholinesterase, antidiabetic and cytotoxic activities of *F. lutea* extracts and the mixture (**1a** + **1b**).

Samples	DPPH Assay ^a^	ABTS^+^ Assay ^a^	Antiacetylcholinesterase Activity ^a^	Antidiabetic Activity ^b^	Cytotoxic Activity ^a^
Ethyl acetate extract	12.8 ± 1.3	184.0 ± 7.0	641.0	54.1	>100
*n*-Butanol extract	26.0 ± 1.8	300.0 ± 5.0	639.5	52.1	40
Mixture ( **1a** + **1b**)	nd ^c^	nd ^c^	na ^d^	na ^d^	>100
Vitamin C	4.4 ± 0.1	4.1 ± 0.1			
Eserine			0.018		
Acarbose				30	
Doxorubicin					0.1

^a^: IC_50_ (µg/mL); ^b^: percent inhibition at 20 µg/mL; ^c^: nd: not determined; ^d^: not active at 1,000 µg/mL.

### 2.5. Antiacetylcholinesterase Activity

The antiacetylcholinesterase activity of the *F. lutea* flower extracts was tested (Table 4). The ethyl acetate and *n*-butanol extracts exhibited weak anti-AChE activity, with IC_50_ values of 641.0 µg/mL and 639.5 µg/mL, respectively. To our knowledge, no study of the anti-acetylcholinesterase activity of *F. lutea* or other *Ferula* species exists in the literature. Compounds **1a** and **1b** were also evaluated and were considered not active at 1000 µg/mL.

### 2.6. Antimicrobial Activity

The antimicrobial activities of *F. lutea* flower extracts against microorganisms was examined in the present study and their potency were qualitatively and quantitatively assessed by the corresponding MIC and MBC values. We did not have enough material to evaluate the antimicrobial activities of the mixture **1a** and **1b**. The results are given in Table 5 and Table 6. The ethyl acetate and *n*-butanol extracts had substantial antimicrobial activity against four bacteria and four Candida yeasts. The *n*-butanol extract (MIC and MBC: 1.25–2.5 mg/mL) exhibited more interesting antimicrobial activities than the ethyl acetate extract (MIC and MBC: 10.00 mg/mL), being especially active against *Staphylococcus aureus* with a MBC and MIC value of 1.25 mg/mL.

**Table 5:** Antibacterial activity of *F. lutea* extracts.

Microorganisms	Ethyl Acetate		*n*-Butanol		Thymol	Gentamecin
	MIC	MBC	MIC	MBC	MIC	MBC
*Staphylococcus aureus* ATCC 25923	10.00	10.00	1.25	0.02	0.20	0.15
*Enterococcus faecalis* ATCC 29212	10.00	>10.00	1.25	0.01	0.60	0.01
*Pseudomonas aeruginosa* ATCC 27853	10.00	10.00	1.25	0.50	1.00	0.50
*Escherichia coli* ATCC 25922	10.00	>10.00	2.50	0.01	0.25	0.01

MIC: Minimum inhibitory concentration (mg/mL); MFC: Minimum bactericidal concentration (mg/mL); Positive control Gentamecin and thymol (µg/mL).

**Table 6:** Anticandida activity of *F. lutea* extracts.

Microorganisms	Ethyl acetate		*n*-Butanol		Thymol	Amphotericin B
	MIC	MFC	MIC	MFC	MIC	MFC
*Candida glabrata* ATCC 90030	0.63	1.25	10.00	10.00	0.16	0.50
*Candida parapsilosis* ATCC 22019	0.30	0.63	10.00	10.00	0.32	0.50
*Candida albicans* ATCC 90028	0.30	0.63	10.00	10.00	0.13	0.50
*Candida kreseui* ATCC 6258	0.30	0.63	10.00	10.00	0.32	0.50

MIC: Minimum inhibitory concentration (mg/mL); MFC: Minimum fungicidal concentration (mg/mL). Positive control amphotericin B and thymol (µg/mL).

Concerning *Candida* yeasts, the ethyl acetate extract (MIC and MBC: 0.30–1.25 mg/mL) exhibited more interesting activities than the *n*-butanol extract (MIC and MBC: 10.00 mg/mL). The ethyl acetate extract was especially active against *C. parapsilosis*, *C. albicans* and *C. kreseui*, with a MFC value of 0.625 mg/mL and MIC value of 0.3 mg/mL; *C. glabrata* was found to be the most resistant species, with MIC of 0.625 mg/mL and MFC of 1.25 mg/mL, compared to the positive control, thymol (MIC value of 0.16 µg/mL). No data on the antimicrobial activity of *F. lutea* or other *Ferula* spp. extracts is available.

### 2.7. Antidiabetic Activity

The inhibitory activity of *F. lutea* extracts against α-amylase is shown in Table 4. The ethyl acetate and *n*-butanol extracts showed very good *α-*amylase inhibitory activity. At 20 μg/mL of extracts, the inhibitory activity was 54.1% and 52.1% for the ethyl acetate and *n*-butanol extracts, respectively. The mixture of compounds **1a** and **1b** was poorly active (IC_50_ > 100 µg/mL). Acarbose is less active than these two extracts. To our knowledge, no previously study on the antidiabetic activity of any *Ferula* species has been reported*.*

### 2.8. Allelopathic Potential

**Figure 3:**
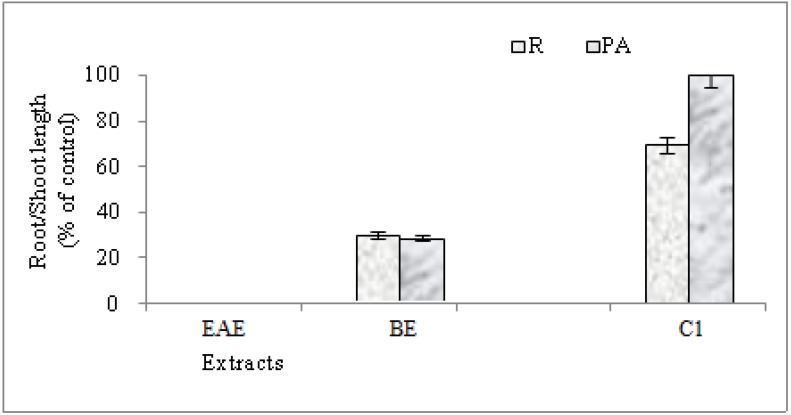
Roots (R) and shoots length (PA), expressed in percent of control, of lettuce in presence (1 mg/mL) of (EAE), *n*-butanol extract (BE) and two diastereoisomers (**1a** and **1b**) (C1) of *F. lutea*; 7 days after germination.

**Figure 4:**
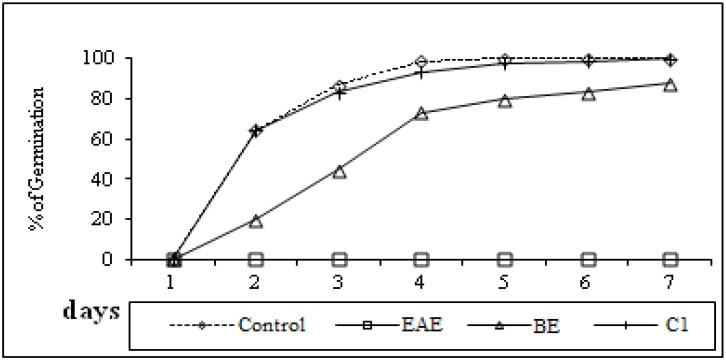
Germination of lettuce seeds in the presence (1 mg/mL) of distilled water (control), ethyl acetate (EAE), n-butanolic extract (BE) and two diastereoisomers (**1a** and **1b**) (C1) of *F. lutea*.

Methanol, in which the residues were dissolved, had no effect on germination hence any observed effects could be attributed to allelochemicals present in the organic extracts and compounds. Bioassays in the presence of extracts showed that the ethyl acetate extracts were the most effective. At the seventh day, the maximum toxicity of these extracts was registered, whereby the germination percentage was 0% (Figure 3). In the others cases, the maximum germination percentage values were 100% for the control and the *n*-butanol extract, but 87% for the mixture of compounds **1a** and **1b**(Figure 4).

For seedling lettuce growth, we recorded a strong activity for the ethyl acetate extract followed by the butanolic extract (BE), then compounds **1a** and **1b** (Figure 4). Indeed, we registered a total inhibition of root and shoot growth in presence of the ethyl acetate extract of *Ferula* plants (Figure 4). Relative toxicity of organic extracts is reported in the literature [27] where a higher phytotoxicity of methanol solutions of ethyl acetate extract is observed on lettuce compared to the *n*-butanol and water fractions. The result suggests that more phytotoxic substances were present in the ethyl acetate fraction than in the butanol one, resulting in more inhibitory effects on the test plant. Allelopathic potential of *Ferula* species is not cited in the literature.

### 2.9. Cytotoxicity Evaluation

The results of the anticancer test on the isolated compounds, ethyl acetate and *n*-butanol extracts using the cancer cell Line, K562 (leukemia cell line) are given in Table 4. The cytotoxicity of the *n*-butanol extract was superior to that of all samples tested with an IC_50_ = 40 µg/mL, while the ethyl acetate extract and compounds **1a** and **1b** were poorly active (IC_50_ > 100 µg/mL). The value of the cytotoxic activity of the n-butanol extract and is good, but not very high compared with the standard substance (doxorubicin).This is the first work citing the cytotoxic activity of *F. lutea* or other *Ferula* spp.

## 3. Experimental

### 3.1. General Experimental Procedures

^1^H- (300 MHz), ^13^C- (75 MHz) and 2D NMR spectra of compounds **1a** and **1b** were recorded in CDCl_3_ with a Bruker NMR-300 spectrometer. The residual solvent resonances were used as internal references. Chemical shifts are expressed in ppm and coupling constants are given in Hertz.

### 3.2. Collection of Plant Material

*F. lutea* flowers were collected in the region of Béja (Tunisia) on April 2010 and identified by Professor Féthia Harzallah Skhiri, at the Laboratory of Genetic Biodiversity and Valorisation of Bioresources of the Higher Institute of Biotechnology of Monastir, Tunisia. A voucher specimen (F.L.F-10) was deposited in the same laboratory.

### 3.3. Extraction and Isolation

The fresh flowers of *F. lutea* (5.8 kg) were macerated at room temperature with MeOH–H_2_O (7:3, 25 L) at room temperature for 7 days and then concentrated by rotary evaporation leaving an oil layer (5.8 g) and an aqueous residue (only the organic solvent was evaporated at the low temperature used ‒30 °C) which was partitioned successively with ethyl acetate and *n*-butanol extracts, yielding, after evaporation of the solvents, the corresponding ethyl acetate (56 g) and *n*-butanol (220 g) extracts. From the all extracts, only the oil layer (5 g) was subjected to silica gel column chromatography eluting successively with pentane–ethyl acetate (95:5 to 100% ethyl acetate) to afford 14 fractions. The ninth subfraction (2.4 g) was chromatographed on silica gel eluting with pentane–ethyl acetate (7:3) to afford a mixture of compounds **1a** and **1b** (2 g) that was difficult to separate. The ^1^H- and ^13^C-NMR spectral data of two diastereoisomers compounds (**1a**, **1b**) are listed in Table 3.

### 3.4. Determination of Total Polyphenol Contents

The amount of total phenolic compounds was determined according to the method of Velioglu *et al.*, [28] which uses the Folin-Ciocalteu reagent. Tested samples were prepared at a concentration of 1 mg/mL. The sample (100 µL) was transferred into a test tube and Folin-Ciocalteu reagent (750 µL, previously diluted 10-times with deionised water) were added and mixed. The mixture was allowed to stand at a temperature of 25 °C for 5 min, then saturated sodium carbonate (Na_2_CO_3_) solution (750 µL) was added to the mixture and then gently mixed. After standing at 25 °C for 90 min, the absorbance was read at 725 nm using an UV–Visible spectrophotometer. A standard curve of gallic acid was used. Total phenolic content of plant parts was expressed as mg gallic acid equivalents per gram of dry weight (mg GAE*/*g DW) through the calibration curve with gallic acid. The calibration curve range was 0–250 μg*/*mL (*R*^2^ = 0*.*99).

### 3.5. Estimation of Total Flavonoid Contents

The AlCl_3_ method [29] was used to determine the total flavonoid content of the sample extracts. Each extract (1.5 mL) was added to equal volumes of a solution of 2% AlCl_3_·6H_2_O (2 g in 100 mL methanol). The mixture was vigorously shaken, and absorbance at 367 nm was read after 10 min of incubation. Total flavonoid content was expressed as mg quercetin/g dry weight (mg QE*/*g DW), through the calibration curve of quercetin. The calibration curve range was 0–50 μg*/*mL (*R*^2^ = 0*.*99). 

### 3.6. Antioxidant Activity by DPPH Assay

Diluted methanol solutions of the samples at different concentrations (1 mL each) were mixed with 1 mL of a freshly prepared (80 µg/mL) methanol solution of DPPH. The resulted solution was incubated at 37 °C for 30 min after what the absorbance of the solution was measured at 517 nm [30]. Lower absorbance of the reaction mixture indicates higher free radical scavenging activity. Tests were carried out in triplicate. Decrease in absorption induced by the tested sample was compared to that of the positive control ascorbic acid. The capability to scavenge the DPPH^.^radical was calculated using the following equation:

Inhibition ratio (DPPH^.^ scavenging effect) (%) = [(A_control_ − A_sample_) / A_control_] × 100 (1)

The IC_50_ was calculated using the linear relation between the compound concentration and the probability of the percentage of DPPH inhibition.

### 3.7. Antioxidant Activity by ABTS Assay

ABTS was dissolved in water to a 7 mM concentration and the ABTS radical cation was produced by adding potassium persulfate to a final concentration of 2.45 mM [31]. The radical generation was completed in the dark at room temperature for 12 h. This solution was then diluted with methanol to adjust its absorbance at 734 nm to 0.706 ± 0.009. To determine the scavenging activity, 1 mL of diluted ABTS^+.^ solution was added to 1 mL of methanolic solutions of extract at different concentrations and the absorbance at 734 nm was measured 10 min after the initial mixing, using methanol as the blank. The percentage of inhibition was calculated by the equation:

[(A_control_ − A_sample_)/A_control_] × 100 (2)

where A_control_ is the absorbance of the control reaction containing all reagents except the tested sample, and A_sample_ is the absorbance of the test compound. Tests were carried out in triplicate. Ascorbic acid was used as the positive control. The IC_50_ was calculated using linear relation between the compound concentration and probability of the percentage of ABTS inhibition.

### 3.8. Antiacetylcholinesterase Activity

Briefly, 50 mM Tris–HCl buffer (100 µL, pH 8), the extract (50 µL) and acetylcholinesterase solution (10 µL) containing 0.26 U/mL were mixed in a microwell plate and left to incubate for 15 min. Subsequently, a solution of AChI (0.023 mg/mL, 50 µL) and 3 mM DTNB (140 µL) were added. The absorbance was read at 405 nm when the reaction reached equilibrium [32]. Eserine was used as a positive control and water served as a negative control and it was considered 100% activity. The percentage inhibition ((%) IP) is given as follows:

(%) IP = 100 − (A_sample_/A_control_) × 100 (3)

where A_control_ is the absorbance of the control reaction containing all reagents except the tested sample, and A_sample_ is the absorbance of the test compounds. Tests were carried out in triplicate and a blank with Tris-HCl buffer instead of enzyme solution was used.

### 3.9. Cytotoxic Activity

Cytotoxic activity of extracts against the human chronic myelogenous leukaemia cell line K562 was estimated by the 3-(4,5-dimethylthiazol-2-yl)-2,5-diphenyltetrazolium bromide (MTT) assay of Khlifi *et al.* [33]. The cells were grown in RPMI- 1640 medium supplemented with 10% faetal calf serum (Gibco, Gaithersburg, MD, USA), air and 5% CO_2_. The resulting blue formazan product can be measured spectrophotometrically. The MTT colorimetric assay was performed in 96-well plates. Adherent human chronic myelogenous leukaemia K562 cells were seeded in a 96-well plate at a concentration of 10^4^ cells/well and incubated at 37 °C overnight in a 5% CO_2_ enriched atmosphere. Cells in exponential growth phase were incubated at 37 °C for 72 h with each tested compound at concentrations ranging from 3.12 to 400 μg/mL. After that, the medium was removed and cells were treated with MTT solution (100 μL, 0.2 mg/mL in PBS) at 37 °C for 2 h. MTT solution was then discarded and DMSO (500 μL) was added to dissolve insoluble formazan crystals. Optical density was measured at 540 nm. Each extract concentration was tested in triplicate. Doxorubicin was used as positive control.

### 3.10. GC-MS Analysis

The mass spectra of compounds **1a** and **1b** (diluted to 1% in the hexane) was achieved by coupled GC/MS analysis, on a system composed of a gas (HP-5890 II, Hewlett Packard Corporation, Palo Alto, California, CA, USA) coupled to a quadrupolar mass spectrometer (HP-MSD 5972 TO) operated in electron impact mode. The column used was an HP polar INNOWAX column of 30 m, 0.25 mm, and 0.25 μm film thickness. Its stationary phase was constituted of polyethylene glycol.

### 3.11. Antidiabetic Activity

Porcine pancreatic α-amylase inhibitory activity was determined using a literature method [34]. Starch azure (2 mg) was used as substrate and was suspended in 0.5 M Tris-HCl (pH 6.9, 0.2 mL) containing 0.01 M CaCl_2_. The tubes containing substrate solution were boiled for 5 min. After 5 min of preincubation at 37 °C, extract dissolved in DMSO (0.2 mL) was added to the tube containing the substrate solution. A volume of porcine pancreatic amylase in Tris-HCl buffer (0.1 mL) was added and then incubated at 37 °C for 10 min and the reaction was stopped by adding 50% acetic acid (0.5 mL). The reaction mixture was centrifuged at 3,000 rpm for 5 min at 4 °C and the absorbance was measured at 595 nm.

### 3.12. Antimicrobial Activity: Determination of Minimum Inhibitory (MIC), Minimum Bactericidal (MBC), and Minimum Fungicidal Concentrations (MFC)

The test microorganisms included the following Gram-positive bacteria: *Staphylococcus aureus ATCC 25923*, *Enterococcus faecalis ATCC 29212*; Gram-negative bacteria: *Escherichia coli ATCC 25922* and *Pseudomonas aeruginosa ATCC 27853* and four *Candida* yeasts: *Candida albicans ATCC 90028*, *C. glabrata ATCC 90030*, *C. kreusei ATCC 6258* and *C. parapsilosis ATCC 22019*).

The MIC values for the antibacterial and anticandidal screening were determined with the broth dilution method (microdilution using 96-well microplates) following the procedure described by Cintia *et al.* [35]. Briefly, all samples were prepared at a concentration of 10 mg/mL by dissolution of the extracts in 10% DMSO. The final concentrations of the plant samples tested ranged from 40 to 2 × 10^4^ µg/mL. The MIC of each sample was defined as the lowest concentration of oil that inhibited either the bacterial or candidal growth, after incubation at 37 °C for 18 to 24 h. The MBC and the MFC were determined by subculture on blood agar at 37 °C for 18 to 24 h. Thymol and gentamicin or thymol and amphotericin B were used as positive controls against the bacterial or candidal strains, respectively. The highest activities for each sample are presented in Table 4 and Table 5 with the most noticeable antimicrobial activities against different strains shown in bold face. All tests were performed in triplicate.

### 3.13. Allelopathic Potential

The two samples concentrated from the ethyl acetate (EAE) and *n*-butanol (BE) extracts and the mixture (**1a** + **1b**) (C1) were again dissolved in MeOH to compare their phytotoxic effects. Five mL of each organic fraction at 6,000 ppm and mixture (**1a** + **1b**) at 1,000 ppm were placed in a Petri dish lined with a sheet of Whatman No. 1 filter paper and evaporated to dryness for 24 h at 24 °C. After evaporation, distilled water (5 mL) was pipetted onto the filter paper. Methanol and distilled water were both were used as negative control. Seeds of lettuce were surface sterilized by immersing in 0.525 mg/mL sodium hypochlorite for 15 min, and then were rinsed four times with deionised water, imbibed in deionised water at 22 °C for 4 h, and carefully blotted using a folded paper towel. Lettuce has been used as tested plant because it is very sensitive to chemicals at low concentration, although it sometimes overestimates the actual allelopathy [36]. Thirty swollen seeds were evenly placed on filter paper wetted with sample in each Petri dish. The germination was recorded at 24-h intervals till 144 h. A seed was considered germinated when the radical protruded ≥2 mm [37]. Data were transformed to percent of control for further analysis. At 7th day after sowing, germination, shoot and root length of target species seedlings were measured. Data were transformed to percent of control for analysis.

## 4. Conclusions

*F. lutea* flowers were investigated for their chemical composition. Total phenols and flavonoids contents of extracts were determined and two diastereoisomers were isolated and identified. To the best of our knowledge, we report the first study on the antiacetylcholinesterase, antidiabectic, antimicrobial, allelopathic and cytotoxic activities of *F. lutea* flower constituents. We can conclude that the ethyl acetate extract has the highest antioxidant activity, the *n*-butanol one possesses an interesting cytotoxicity and finally both extracts have good antidiabetic activity. Nevertheless, it is interesting to continue the fractionation work to isolate the molecules responsible for the antioxidant, antidiabetic and cytotoxic activities. Compounds **1a** and **1b** were isolated as a mixture and their activities were evaluated for the first time.
